# Complete genome sequence data of *Flavobacterium anhuiense* strain GSE09, a volatile-producing biocontrol bacterium isolated from cucumber (*Cucumis sativus*) root

**DOI:** 10.1016/j.dib.2019.104270

**Published:** 2019-07-15

**Authors:** Jin-Ju Jeong, Siti Sajidah, Ji Yeon Oh, Mee Kyung Sang, Kyoung-Su Kim, Ki Deok Kim

**Affiliations:** a Laboratory of Plant Disease and Biocontrol, Department of Biosystems and Biotechnology, Korea University, Seoul, South Korea; b Division of Agricultural Microbiology, National Academy of Agricultural Science, Rural Development Administration, Wanju, South Korea; c Division of Bioresource Sciences, Bioherb Research Institute, Kangwon National University, Chuncheon, South Korea

**Keywords:** Biocontrol, Complete genome sequence, EggNOG analysis, *Flavobacterium anhuiense*, *Phytophthora capsici*

## Abstract

*Flavobacterium anhuiense* (previously identified as *Flavobacterium johnsoniae*) strain GSE09 is a volatile-producing bacterium that exhibits significant biocontrol activity against an oomycete pathogen, *Phytophthora capsici*, on pepper plants. Here, we report the complete genome sequence data of strain GSE09, isolated from surface-sterilized cucumber root. The genome consists of a circular 5,109,718-bp chromosome with a G + C content of 34.30%. A total of 4,138 complete coding sequences including 15 rRNA, 66 tRNA, 3 ncRNA, and 51 pseudogene sequences were retrieved. Thus, the genome sequence data of *F. anhuiense* GSE09 may facilitate the elucidation of many biological traits related to the biocontrol against plant pathogens.

**Table:** Specification table

Subject area	Biology
More specific subject area	Microbiology and Genomics
Type of data	Complete genome sequence data of *Flavobacterium anhuiense* GSE09
How data was acquired	Genome sequencing using PacBio RS II at Theragen Etex Bio Institute, Suwon, South Korea
Data format	Raw and analyzed data
Experimental factors	DNA was extracted from *F. anhuiense* GSE09
Experimental features	Whole genome sequencing, assembly, and annotation.
Data source location	*F. anhuiense* GSE09 was isolated from the surface-sterilized root of a cucumber plant grown in a field in Gunsan, Korea.
Data accessibility	The genome sequence of *F. anhuiense* GSE09 has been deposited in DDBJ/ENA/GenBank under the accession number CP016907 (https://www.ncbi.nlm.nih.gov/nuccore/CP016907)
Related research	M.K. Sang, K.D. Kim, The volatile-producing *Flavobacterium johnsoniae* strain GSE09 shows biocontrol activity against *Phytophthora capsici* in pepper. J. Appl. Microbiol. 113 (2012) [1]

**Table:** 

**Value of the data** • The genome data of *F. anhuiense* GSE09 may be helpful in understanding biological traits such as plant resistance and plant growth promotion related to the biocontrol against plant pathogens. • In *F. anhuiense* GSE09 genome, 61 defense mechanism genes and 5 cell motility genes were identified, which could possibly be significant traits of its biocontrol activity. • The *F. anhuiense* GSE09 genome data will provide valuable information to reveal the phylogenetic diversity of the *Flavobacterium* species.

## 1 Data

**Fig. 1:**
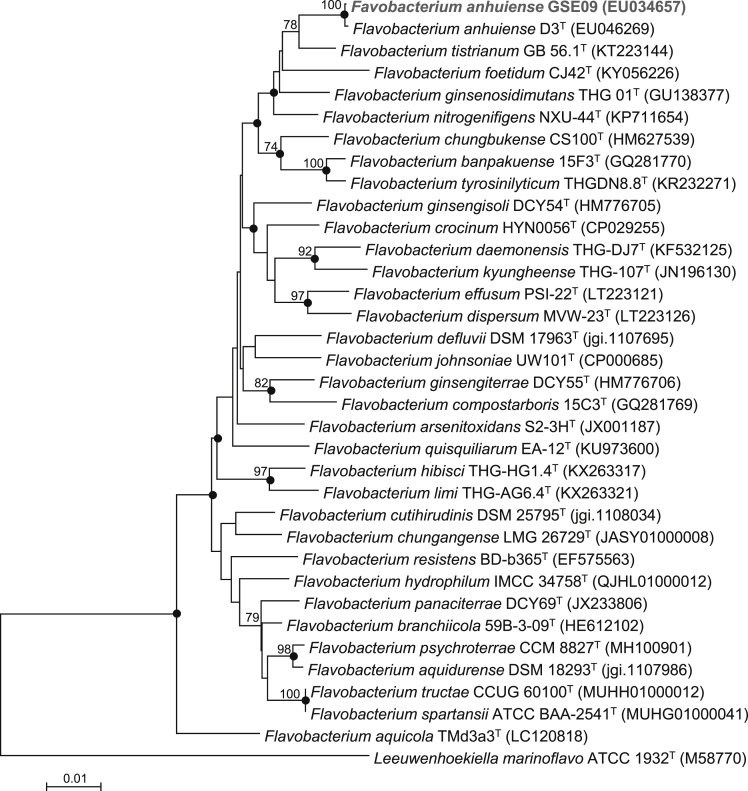
Neighbor-joining phylogenetic tree, based on 16S rRNA gene sequences, showing the relationship between strain GSE09 (GenBank accession no. EU034657) and other species of the genus *Flavobacterium*. Filled circles on the branching points indicate that the corresponding nodes were also recovered in trees constructed using the maximum-likelihood and maximum-parsimony algorithms. Bootstrap values (≥70%) of 1000 analyses are shown at the branch points. *Leeuwenhoekiella marinoflavo* ATCC 1932^T^ was used as an outgroup. Bar, 1 nt substitution per 100 nt of the 16S rRNA gene sequence.

Previous studies [1], [2], [3] showed that strain GSE09, isolated from the surface-sterilized root of a cucumber (*Cucumis sativus*) plant grown in a field in Gunsan, South Korea in 2002, significantly inhibited infections by a soilborne oomycete pathogen, *Phytophthora capsici*, the causal agent of Phytophthora blight of pepper. This strain produces the volatile organic compound (VOC), 2,4-di-*tert*-butylphenol, which inhibits mycelial growth, sporulation, and zoospore germination of the pathogen [1,3]. In a previous study [1], strain GSE09 was identified as *Flavobacterium johnsoniae*; however, it has now been re-identified as *Flavobacterium anhuiense* based on phylogenetic analysis of the 16S rRNA gene sequences (Fig. 1).

**Fig. 2:**
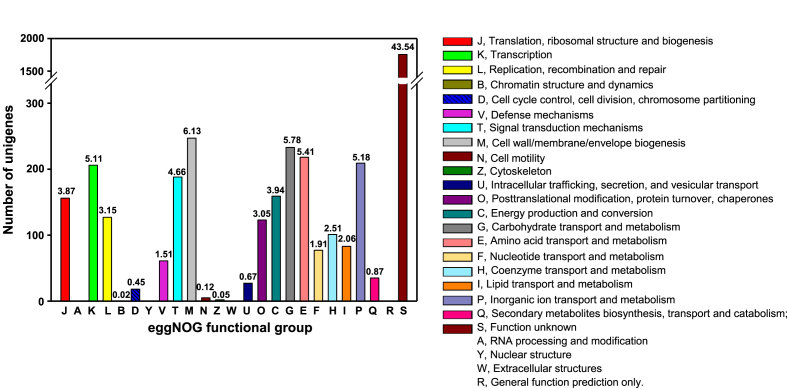
Number and percentage of *Flavobacterium anhuiense* strain GSE09 unigenes assigned to each functional group using the evolutionary genealogy of genes: non-supervised orthologous groups (eggNOG) analysis. The number on the bar represents the percentage of unigenes assigned to each functional group out of the total numbers of unigenes.

The genome of strain GSE09 consists of a circular 5,109,718-bp chromosome with a G + C content of 34.30%. In addition, 4,138 complete coding sequences (CDSs) were predicted, out of which 15 rRNA, 66 tRNA, 3 ncRNA, and 51 pseudogene sequences were retrieved. Moreover, 2,276 unigenes (56.46%) out of a total of 4,031 unigenes in GSE09 were assigned to 20 out of 24 functional groups based on the evolutionary genealogy of genes: non-supervised orthologous groups (eggNOG) analysis (Fig. 2). A total of 247 (6.13%), 233 (5.78%), and 218 (5.41%) unigenes were assigned to the ‘cell wall/membrane/envelope biogenesis’, ‘carbohydrate transport and metabolism’, and ‘amino acid transport and metabolisms’ groups, respectively; however, no unigenes were assigned to the ‘RNA processing and modification’, ‘nuclear structure’, ‘extracellular structures’, and ‘general function prediction’ groups. Furthermore, 61 unigenes were assigned to ‘defense mechanisms’ and 5 were assigned to ‘cell motility’, which may be related to the biocontrol traits of the strain (Fig. 2).

The genome of strain GSE09 also contained genes related to colonization (*e.g.*, motility protein B, swarming motility protein SwrC, and gliding motility lipoprotein GldH precursor) [4], [5], [6] and antibacterial activity (*e.g.*, thiazole synthase and polyketide cyclase/dehydrase and lipid transport) [7], [8]. Furthemore, it contained various genes related to plant defense against plant pathogens and genes related to plant growth promotion, such as ammonia, siderophores, and indole-3-acetic acid (IAA) [9], [10], [11].

## 2 Experimental design, materials and methods

Strain GSE09 was re-identified using phylogenetic analysis of the 16S rRNA gene sequence data (accession no. EU034657) in NCBI [1]. The analysis was performed with strain GSE09 and type strains of species belonging to the genus *Flavobacterium* using the molecular evolutionary genetics analysis (MEGA) version 6 software. Phylogenetic trees were constructed using neighbor-joining, maximum-likelihood, and maximum-parsimony algorithms [12], [13], [14]. The tree topology was confirmed via 1000 replications of bootstrap analysis [15].

The genomic DNA of strain GSE09, which was prepared as described previously [1], was extracted using the DNeasy Plant Mini Kit (Qiagen, Valencia, CA, USA) according to the manufacturer's protocols. Genome sequencing of the strain was performed using the PacBio RS II instrument (Pacific Bioscience, Menlo Park, CA, USA) at the Theragen Etex Bio Institute (Suwon, South Korea). Single-molecule real-time (SMRT) sequencing with PacBio RS II was conducted with SMRTbell DNA template libraries that were generated using the standard 20-kb library protocols of the PacBio SMRTbell template prep kit (Pacific Bioscience). The sequence reads generated were used for *de novo* assembly using the PacBio Hierarchical Genome Assembly Process version 3 (HGAP3), which were then polished using Quiver [16]. Genome annotation of strain GSE09 was performed using the Prokaryotic Genome Annotation Pipeline (PGAP) service provided by NCBI. Furthermore, the eggNOG analysis of strain GSE09 genes was conducted using CLgenomics ver. 1. 53 [17].
